# A Laudable Ambition, etc.

**Published:** 1888-02-11

**Authors:** 


### THE NURSING MIRROR.

Communications for this column should be addressed The Mirror, care of the Editor, 38, Old Jewry, London, E.C.

### A LAUDABLE AMBITION.

I AM glad to see that someone has taken up the question of certificates for nurses. I feel sure there are many who would be very glad to be able to get them, even if it did give them some little trouble and expense. In these days of competition a certificate is necessary if a nurse is ever to be promoted, and surely that is the ambition of a great many? Their employers could speak for their practical knowledge, while *The Hospitals Association*, by having an examination, could find out about the theory. Then, if they are satisfied that there is a good knowledge of both, a certificate might be granted; and if there are many uncertificated nurses situated like myself, they would think it a great boon. At present there is only one way of getting a certificate; that is, by going into hospital as a probationer for a year, and paying a guinea a-week. Surely these are rather hard lines for experienced nurses who have been in the work for years, and have valuable experience both in hospital, private, and district nursing that many certificated nurses have not. I have been an interested reader of THE HOSPITAL ever since it was started, but this question of certificates for the deserving non-certificated is interesting me deeply, for it seems to open out a valuable sphere of usefulness and good work second only to the "Pension Fund" itself.

AN UNCERTIFICATED NURSE.

